# A Clinical Introduction to the Practice of Auscultation, and Other Modes of Physical Diagnosis; Intended to Simplify the Study of the Diseases of the Lungs and Heart

**Published:** 1846-06

**Authors:** 


Art. IX.—*A Clinical Introduction to the Practice of Auscultation, and other modes of Physical Diagnosis; intended to Simplify the Study of the Diseases of the Lungs and Heart.* By H. M. Hughes, M,D., Fellow of the Royal College of Physicians; Assistant Physician to Guy's Hospital, &c. Philadelphia: Lea & Blanchard. 1846. 12mo. pp. 270.

We have just noticed the work of Dr. Gerhard on a subject kindred to the one of which the little volume of Dr. Hughes treats; but while that work embraces the treatment, as well as the diagnosis and symptoms of thoracic diseases, this is confined to these latter points. Its aim is to instruct the student how he shall proceed at the bed-side of patients, whose ailments of the chest he is trying to comprehend. "I know," says the author, "that after the perusal of works especially treating of diseases of the chest, pupils, and practitioners too, are often quite incapable of instituting an ordinary examination, and of distinguishing the more common physical signs. With terms they may be sufficiently familiar; the theory of morbid sounds may not be unknown to them; but how they are to elicit some of these sounds, and how they are to distinguish others, they know not. They cannot, in fact, *practise* auscultation and percussion; and they are unacquainted with the mode of setting about it." It is only by practice that competent knowledge can be attained; but unless one has enjoyed the advantages of clinical instruction, he finds it difficult to take the first step towards the acquisition of such knowledge. This work was written to aid such beginners, and contains directions how auscultation and percussion are to be performed, as well as an account of the indications afforded by them. It has these decided advantages, that it is small, cheap, and intelligible; and written as it is by one who is thoroughly master of his subject, it is confidently recommended to every student or practitioner who needs a guide in his explorations of the Chest. The woodcut which is affixed will give the reader a good idea of the artificial divisions of the chest and abdomen.

